# Seroprevalence of Bovine Brucellosis in Selected Districts of Zambia

**DOI:** 10.3390/ijerph18041436

**Published:** 2021-02-03

**Authors:** Ruth Lindizyani Mfune, Melai Mubanga, Isaac Silwamba, Frederick Sagamiko, Steward Mudenda, Victor Daka, Jacques Godfroid, Bernard M. Hangombe, John B. Muma

**Affiliations:** 1 School of Veterinary Medicine, The University of Zambia, P.O. Box 32379, Lusaka, Zambia; melaimubanga@gmail.com (M.M.); isaacsilwambak@gmail.com (I.S.); freshsteward@gmail.com (S.M.); dakavictorm@gmail.com (V.D.); mudenda68@yahoo.com (B.M.H.); jmuma@unza.zm (J.B.M.); 2 Michael Chilufya Sata School of Medicine, Copperbelt University, P.O. Box 71769, Ndola, Zambia; 3 Maswa District Council, P.O. Box 170, Maswa, Simiyu, Tanzania; fsagamiko@yahoo.co.uk; 4 Department of Arctic and Marine Biology, UiT The Arctic University of Norway, 9037 Tromsø, Norway; jacques.godfroid@uit.no

**Keywords:** cattle, brucellosis, seroprevalence, Zambia

## Abstract

Brucellosis is an infectious zoonosis that has huge economic and public health implications globally. The disease is prevalent in humans, livestock and wildlife in Sub-Saharan Africa. A cross-sectional study was conducted between 6 May 2017 and 31 July 2020 during which 1712 sera from 175 cattle herds in five districts from Southern, Western and Eastern Provinces of Zambia were collected and screened against brucellosis. The Rose Bengal Test (RBT) and competitive Enzyme-linked Immuno Assay (c-ELISA) were used in serial testing for the detection of antibodies against *Brucella* species. A total of 127 animals from 37 herds tested positive, giving overall individual animal and herd-level seroprevalences of 7.53% (95% CI: 6.28–8.78%) and 21.14% (95% CI: 15.0–27.2%), respectively. Namwala district had the highest herd seroprevalence (33.9%, 95% CI: 21.6–46.1%), while Lundazi did not record any seropositivity. Comparably, Southern Province had the highest individual animal (8.97%, 95% CI: 7–11%) and herd-level (28.5%, 95% CI: 20.3–36.7%) seroprevalences, although this was not statistically significant. Within Southern Province, higher seropositivity was observed in Namwala district (OR: 8.55; CI: 2.66–27.44), among female animals (OR: 2.48; CI: 1.38–4.46) and in those aged 11 years and above (OR: 2.67; CI: 1.34–5.34) as well as in gravid cows (OR: 4.34; CI: 2.08–8.92). Seropositivity was also observed among some animals with hygromas (OR: 6.5; CI: 0.45–94.08) and those with a history of abortion (OR: 1.13; CI: 0.18–7.28) although the findings were not statistically significant. *Brucella* seroprevalence among traditional cattle in Zambia remains high. Control programs against bovine brucellosis must be introduced to reduce its impact on human health and animal production.

## 1. Introduction

Brucellosis is an infectious zoonotic disease of economic and public health importance that affects livestock, wildlife and humans worldwide [1]. The disease is caused by a Gram-negative coccobacilli bacterium of the genus *Brucella*, which currently contains 12 host-specific species [2]. Even though *Brucella abortus* is the main cause of brucellosis in cattle, other species have also been isolated [3,4,5]. The associated clinical signs include abortions, infertility, reduced milk production, calf mortality, hygroma, epididymitis and orchitis [6]. Transmission between animals is through contact with aborted fetuses, placentas, vaginal discharges and milk from infected animals. Production losses due to abortions, infertility and sterility in adult animals as well as reduced milk production contribute to serious economic losses in the livestock industry, while humans are at risk of *Brucella* infections through occupational exposure to animals and the consumption of unpasteurized milk [7]. This underscores the public health importance of the disease.

The diagnosis of brucellosis is performed using bacteriological, molecular and serological methods [1]. Bacteriological tests are referred to as the “Gold standard”, as they are highly specific and confirmatory compared to other methods [1,3]. To increase the likelihood of isolating the *Brucella* organisms, the bacteriological samples collected for culture often include milk [8], hygroma fluids [9], fetal materials and vaginal discharges up to six weeks post-abortion or parturition [1,10]. Serology tests detect antibodies directed against epitopes associated with the smooth lipopolysaccharide (S-LPS). The common serology tests used are the Rose Bengal Test (RBT), Serum Agglutination Test (SAT), Compliment Fixation Test (CFT), Milk Ring Test (MRT) and Enzyme-linked Immuno Assay (ELISA) [11]. These tests are recommended by the World Organization for Animal Health (OIE) [1]. In brucellosis serodiagnosis, serial testing is recommended to improve the specificity of the test regime [10]. The common serial test combination uses RBT and cELISA for screening and confirmation, respectively.

Most developed countries have managed to eradicate bovine brucellosis [12]. However, it is still endemic in most Sub-Saharan African countries with varying seroprevalence rates [13,14,15,16,17,18]. In Southern Africa, the accurate brucellosis picture is not clearly known because most reports are based on non-representative laboratory results. In South Africa, for instance, a 5.5% seroprevalence reported recently [19] showed that the disease control scheme introduced in 1968 had an impact in reducing the disease prevalence. In Namibia, brucellosis is endemic in communal herds and commercial farms in low proportions [20]. In Zimbabwe, despite the implementation of a brucellosis vaccination program, the disease prevalence varies across provinces, with the latest report indicating a 30.1% herd seroprevalence [18]. In Angola, a 40.1% seroprevalence was reported in cattle herds [21], while in Malawi, a seroprevalence of 7.7% was reported in dairy cattle in the northern region that borders the Eastern Province of Zambia [22].

In Zambia, a few studies that were conducted in wildlife and cattle in Southern and Western Provinces almost eight years ago estimated the disease seroprevalence in smallholder and traditional cattle [23,24,25,26], while the status is unknown in Eastern Province and other parts of the country. Therefore, this study aimed to estimate the seroprevalence of bovine brucellosis in Southern, Western and Eastern Provinces to fill the knowledge gap for the current epidemiological status of the disease in the high-cattle-farming areas of Zambia. Such information is vital in mapping and understanding the epidemiological distribution of the disease, which can be used in the control of brucellosis in Zambia and other counties in the region with similar settings.

## 2. Materials and Methods

This cross-sectional study was carried out in five districts that were purposively selected from three provinces of Zambia, namely, Namwala, Choma and Monze (Southern Province), Senanga (Western Province) and Lundazi (Eastern Province). This is because they have higher cattle populations in Zambia compared to other provinces. The traditional cattle breeds that are reared in the study areas are, namely, Tonga, Barotse and Angoni in Southern, Western and Eastern Provinces, respectively. Southern Province lies between latitudes 15°14′ S and 17°42′ S and longitudes 25° E and 28° S. It has a total land surface area of 85,283 km^2^, an estimated human population of 1,907,784 [27] and a cattle population of 2,105,891 [28]. A pastoral or nomadic cattle-grazing system is practiced, where animals are grazed in the Kafue flats/floodplains in dry seasons and moved to the upper areas during the wet season [24]. Western Province lies between latitudes 14° S and 17° S and longitudes 22° E and 25° E andhas a total land area of about 126,386 km^2^, a human population of 1,007,855 [27] and an estimated cattle population of 890,288 [28]. Western Province has dominant sandy soils and the Barotse floodplain of the Zambezi River, which naturally waters the grasslands. Over three quarters of the cattle in Western Province are pastured in the floodplains. They are managed under a transhumance system, whereby they are moved between the floodplains (January to July) and adjacent uplands (the rest of the year) [29]. Pastoral livestock farming is the mainstay of the province’s economy, followed by fish and crop farming.

The required sample size was calculated using the formula n = z^2^ ∗ p ∗ (1 − p)/d^2^, where z = 1.96, p = the expected herd seroprevalence of 32% [14], d = the desired absolute precision of 10% and confidence level of 95%. The resulting sample size of 84 was multiplied by the design effect (D) of 1.9, calculated using the formula D = 1 + (b − 1) roh [30]. The average number of samples per cluster (b) was 10, and the intracluster correlation coefficient or rate of homogeneity (roh) was 0.1 [31]. The calculated sample size was 160 herds. To account for non-response, a 10% adjustment was made, bringing the required herd size to 176.

A total of 1712 cattle from 175 herds were randomly sampled from five districts, namely, Namwala, Choma and Monze (Southern Province); Senanga (Western Province); and Lundazi (Eastern Province). This was performed by determining the sampling interval based on the number of herds available and the required sample size.

Five milliliters of blood was aseptically collected from the jugular vein into labelled sterile plain vacutainer tubes. In the field, the serum was separated using a portable field centrifuge and stored in pre-labelled cryovial tubes at −20 °C until transportation to the University of Zambia for laboratory analysis. All the serum samples were screened for Brucella spp. antibodies using The Rose Bengal test kit (Central Veterinary Laboratory, New Haw, Addelestone Surrey KT153NB, London, UK) according to the test procedure recommended by the OIE Terrestrial Manual [1]. A sample was considered positive if any visible sign of agglutination was observed. All positive sera were retested using the *cELISA test kit* (INGEZIM BRUCELLA COMPAC 2.0, Madrid, Spain) as per the manufacturer’s instructions and as described by others [14]. A sample was considered positive for brucellosis if it tested positive on both the RBT and cELISA.

Data were entered into a spreadsheet (Microsoft Excel 2010 version, Redmond, WA, USA), and the proportions were estimated for the individual and herd *Brucella* prevalence. The individual animal seroprevalence was calculated by dividing the number of RBT- and c-ELISA-positive animals by the total number of animals that were tested. The herd-level seroprevalence was calculated by dividing the number of herds with at least one reactor on the RBT and c-ELISA by the number of all the herds tested. Associations between hypothesized risk factors and the outcome variables were assessed, and statistical analysis was performed using Chi-square tests and logistic regression using the statistical software STATA^®^ version 16 (StataCorp LLC, College Station, TX, USA).

## 3. Results

A total of 129 animals from 37 herds were seropositive, giving overall individual animal (Table 1)- and herd (Table 2)-level brucellosis seroprevalences of 7.53% (95% CI: 6.28–8.78) and 21.14% (95% CI: 15.0–27.2), respectively. Namwala district had the highest individual animal (12.45%, 95% CI: 9.8–15.1) and herd level (33.9%, 95% CI: 21.6–46.1) seroprevalences (Table 1 and Table 2), while Lundazi did not record any seropositivity (Table 1 and Table 2). Southern Province had the highest herd-level (28.5%, 95% CI: 20.3–36.7) (Table 3) and individual animal (8.97%, 95% CI: 7–11) (Table 4) seroprevalences. The animal seropositivity was statistically insignificantly higher in Namwala district (OR: 8.55, CI: 2.66–27.44), among female animals (OR: 2.48, CI: 1.38–4.46), and in those aged 11 years and above (OR: 2.67, CI: 1.34–5.34) as well as in gravid cows (OR: 4.31, CI: 2.08–8.92). Seropositivity was observed among some animals with hygromas (33.3%), a history of abortion (5.6%) and infertility (11.1%) (Table 4), although these results were not statistically significant.

The odds ratios suggested that animals in Namwala were more likely (OR = 8.55, CI: 2.66–27.44) to test positive than those from the other districts. Furthermore, animals aged 11 years and above were more likely (OR = 2.67, CI: 1.34–5.34) to test positive than those from other age groups. On the other hand, gravid cows (OR = 4.31, CI: 2.08–8.92), those with a history of abortion (OR = 1.13, CI: 0.18–7.28) and those with hygroma (OR = 6.5, CI: 0.45–94.08) were more likely to test positive (Table 4).

## 4. Discussion

This study aimed at estimating the seroprevalence of bovine brucellosis in selected districts of Zambia. The overall seroprevalences at the animal and herd levels were 7.53 and 21.14%, respectively. Although we did not detect any seropositivity in Eastern province, Southern Province had the highest herd and individual animal seroprevalences, respectively (28.5 and 8.97%), followed by Western province (10.0 and 8.35%). The individual animal seroprevalence in this study was slightly higher than the 6% reported in smallholder dairy cattle in Lusaka and Southern Provinces [25]. The herd seroprevalence was also comparable to the previously reported 20.7% in Southern Province [26] but lower than the 46.2–74% in the livestock–wildlife interface area of the Kafue flats [24] among traditional cattle in Zambia. The herd seroprevalence was slightly higher in Southern Province and Namwala district despite being statistically insignificant. The odds ratio also suggested that animals in Namwala were more likely (OR = 8.55, CI: 2.66–27.44) to test positive than those from other districts. The observed high seropositivity at the individual animal and herd levels can be attributed to the fact that the Province has the highest traditional cattle population in Zambia, which predominantly relies on communal grazing in the Kafue flood plains [24]. The movement of cattle herds to the plains, in search of greener pastures, is high, which results in interherd interactions and the consequent spread of infection [32]. The slight increase observed in the herd and animal seroprevalences over the decade possibly shows that the disease has become stable over the years, thereby reaching an endemic state. The continuing lack of control measures for brucellosis in Zambia is evidenced by the government’s priorities in disease control programs, where some livestock diseases are considered more important (e.g., foot and mouth disease, East Coast fever and contagious bovine pleuropneumonia) than others [33]. This is leading to unmitigated transmission and has possibly contributed to this plateau state. The traditional cattle sector constitutes a significant proportion of the cattle production system in Zambia [34]; hence, this sustained disease pressure is worrying due to the economic and public health risks it poses to pastoral communities. The reported absence of *Brucella* seropositivity in Lundazi district, Eastern Province, is inconclusive, as it may be attributed to the small sample size; however, it is interesting to note that a seroprevalence of 7.7% was observed in Northern Malawi [22], which borders Lundazi district in Zambia. We recommend a more detailed study in Eastern Province to further investigate the disease epidemiology.

Our herd seroprevalence is comparatively lower than the 25.6, 29.2, 32, 40.1 and 45.9% reported in Ethiopia, Tanzania, Cameroon, Nigeria and Angola, respectively [13,14,16,17,21]. In comparison to findings from studies in other countries in Southern Africa, our individual animal- and herd-level seroprevalences are comparatively lower than the 9.9 and 30.1% reported in Zimbabwe [35]. At the animal level, our findings are higher than the 5.5% reported in South Africa [19], but comparable to the 7.7% in Northern Malawi [22]. The result variations observed may be attributed to several factors including the sampling techniques and sample sizes, the different diagnostic tests and interpretations used, and seasonal cattle movements in search of pastures amidst droughts.

Although statistically not significant, the seropositivity was higher in female animals (8.45%), those aged 11 years and above (14.1%) and gravid cows (13.79%). This agrees with findings from similar studies [13,16,24,35] and is consistent with the known relationship between age, sex and *Brucella* status. Animals aged 11 years and above were more likely (OR = 2.67, CI: 1.34–5.34) to test positive than other age groups. On the other hand, gravid cows (OR = 4.31, CI: 2.08–8.92), those with abortion histories (OR = 1.13, CI: 0.18–7.28) and those with hygroma (OR = 6.5, CI: 0.45–94.08) were more likely to test positive. It has been documented that female and older animals tend to have increased chances of testing positive for Brucellosis [36]. As the animal reaches sexual maturity, the levels of growth-stimulating factors for *Brucella* organisms become high [37], while constant exposure to the *Brucella* organisms increases with age. The high seropositivity observed among females could also be due to the high number of females that were sampled compared to males. The high seroprevalence among pregnant cattle can be explained by the elevated erythritol levels in the placental and fetal fluids during the third trimester [38]. These high sugar levels stimulate the growth and multiplication of the bacteria in the reproductive organs.

The combined use of RBT and cELISA tests serially in our study maximized the specificity of the test system, while reducing labor and costs. The RBT was used as a cheap and easy-to-perform screening test with high sensitivity but limited specificity. To improve the specificity of the test system, only RBT-positive reactors were confirmed by the expensive and laborious but highly sensitive and specific cELISA. Thus, as far as misclassification bias is concerned, the seroprevalence estimates in our study may be assumed to be unbiased.

## 5. Conclusions

The overall brucellosis seroprevalence rates at the individual animal and herd levels were 7.53 and 21.14%, respectively. The seropositivity was higher in female animals, those aged 11 years and above and gravid cows, although this was not statistically significant. It is interesting to note that the *Brucella* seroprevalence seems to have maintained enzootic stability over a decade in Southern and Western Provinces. There is a need to develop and implement multisectoral One Health surveillance and control strategies to minimize the disease burden in animals and, consequently, in humans.

## Figures and Tables

**Table 1:** Individual animal brucellosis seroprevalence by district.

District	n	Positive	Seroprev.%	95% CI
Lundazi	251	0	0	-
Choma	186	3	2.15	0.1–4.2
Monze	326	21	6.44	3.7–9.1
Namwala	602	74	12.45	9.8–15.1
Senanga	347	29	8.35	5.4–11.3
Total	1712	129	7.53	6.28–8.78

Seroprev.—Seroprevalence; %—Percentage; CI—Confidence interval; n—Number.

**Table 2:** Herd-level brucellosis seroprevalence by district.

District	Herd n	Positive	Seroprev.%	95% CI
Lundazi	26	0	0.00	-
Choma	23	2	8.60	3.2–20.6
Monze	37	12	32.4	17.0–47.8
Namwala	59	20	33.9	21.6–46.1
Senanga	30	3	10.0	1.0–20.9
Total	175	37	21.14	15.0–27.2

Seroprev.—Seroprevalence; %—Percentage; CI—Confidence interval; n—Number.

**Table 3:** Herd-level brucellosis seroprevalence by province.

Province	Herd n	Pos.	Herd Seroprev.%	95% CI
Southern	119	34	28.5	20.3–36.7
Western	30	3	10.0	0.1–20.9
Eastern	26	0	0	-
Total	175	37	21.14	15.0–27.2

Seroprev.—Seroprevalence; %—Percentage; CI—Confidence interval; n—Number

**Table 4:** Association between animal characteristics and individual animal-level brucellosis seropositivity assessed by logistic regression.

Variable	Category	Pos./Tested	Seropositivity (%)	Odds Ratio	95% CI
Province	S/P	100/1114	8.97	Ref.	
	W/P	29/347	8.35	1	0.61–1.45
	E/P	0/251	0	1	-
District	Choma	3/186	1.61	Ref.	
	Monze	21/326	6.44	4.2	1.23–14.27
	Namwala	74/602	12.29	8.55	2.66–27.44
	Senanga	29/347	8.36	5.56	1.67–18.52
	Lundazi	0/251	0	1	-
Age	0–5 y	57/985	5.78	Ref.	
	6–10 y	59/649	9.09	1.63	1.12–2.37
	>11 y	11/78	14.1	2.67	1.34–5.34
Sex	Male	13/363	3.58	Ref.	
	Female	114/1349	8.45	2.48	1.38–4.46
Reproductive status	Bull	13/363	3.58	Ref.	
	Cow	61/834	7.31	2.12	1.15–3.91
	Lactating	33/370	8.92	2.63	1.36–5.09
	Pregnant	20/145	13.79	4.31	2.08–8.92
Animal history	Cows with calves < 3 months old	3/42	7.14	Ref.	
	Abortion	25/2	8.0	1.13	0.18–7.28
	Infertility	0/6	0	1	-
	Hygroma	3/1	33.3	6.5	0.45–94.08
	No hygroma	121/1636	7.39	1.04	0.32–3.41

S/P—Southern province; W/P—Western province; E/P—Eastern province; Pos.—Positive; CI—Confidence interval; Ref.—Reference.

## Data Availability

The data supporting the reported results can be made available on request from the corresponding author.
